# Supplementary material for: Clinical and Imaging Outcomes after Vitamin D Supplementation in Patients with Multiple Sclerosis: A Systematic Review
Source: Nutrients. 2023 Apr 18;15(8):1945. doi: 10.3390/nu15081945 (PMC10141047; doi:10.3390/nu15081945)
Supplement: Supplementary file 1 [file nutrients-15-01945-s001.zip › nutrients-2324377-supplementary.pdf]

nutrients MDPI

Table S1. The applied electronic search strategy for the systematic review of databases.

Database	Electronic Search Strategy for the Systematic Review
PubMed	((multiple sclerosis[Title/Abstract] OR clinically isolated syndrome [Title/Abstract]) AND (vitamin D[Title/Abstract] OR vitamin D2[Title/Abstract] OR vitamin D3[Title/Abstract] OR D2[Title/Abstract] OR D3[Title/Abstract] OR ergocalciferol[Title/Abstract] OR cholecalciferol[Title/Abstract] OR 25-hydroxyvitamin D[Title/Abstract] OR 3-epi-25hydroxyvitamin D[Title/Abstract] OR calcitriol[Title/Abstract] OR dihydroxycholecalciferol[Title/Abstract]))<br>Filters applied: Humans
ClinicalTrials.gov	"multiple sclerosis" in condition or disease field and "vitamin D"<br>Filters applied: Interventional Studies (Clinical Trials)
EU Clinical Trials	"multiple sclerosis" AND "vitamin D"

Table S2. Assessment of the risk of bias of the randomized trials selected in the systematic review according to the RoB 2 tool. Green, orange and red colors mean low risk of bias, some concerns and high risk of bias, respectively.

Ref.	First author, year	Randomization process	Time of recruitment	Adhering to intervention	Missing outcome data	Measurement of outcomes	Selection of the reported result	Overall risk of bias
[19]	Dörr et al., 2020	Orange	Green	Green	Orange	Green	Orange	Orange
[18,38]	Hupperts et al., 2019	Orange	Green	Green	Green	Green	Orange	Green
[20]	Camu et al., 2019	Orange	Green	Green	Green	Green	Orange	Green
[22]	O'Connell et al., 2017	Orange	Orange	Green	Orange	Green		Orange
[34]	Sotirchos et al., 2016	Orange	Orange	Green	Orange	Green	Green	Orange
[31]	Etemadifar et al., 2015	Red	Red	Red	Red	Green	Green	Red
[28]	Achiron et al., 2015	Green	Green	Orange	Green	Green	Orange	Green
[29,40]	Golan et al., 2013a and 2013b	Orange	Orange	Orange	Red	Green	Orange	Red
[24,41]	Soilu-Hänninen et al., 2012	Green	Green	Orange	Green	Green	Orange	Green
[25]	Kampman et al., 2012	Orange	Orange	Green	Green	Green	Orange	Orange
[32]	Shaygannejad et al., 2012	Orange	Orange	Green	Green	Green	Orange	Orange
[37]	Stein et al., 2011	Green	Green	Green	Orange	Green	Orange	Green
[33]	Mosayebi et al., 2011	Orange	Green	Orange	Orange	Green	Green	Orange
[35,42]	Burton et al., 2010	Orange	Orange	Red	Orange	Green	Green	Red
